# Mental health vulnerability in multicultural families: Risk factors among homogenous country

**DOI:** 10.1017/gmh.2024.108

**Published:** 2024-10-24

**Authors:** Eunah Kim, Myung-Bae Park

**Affiliations:** 1 Institute of Health and Environment, Seoul National University, Seoul, Republic of Korea; 2 Division of Health Administration, Yonsei University Mirae Campus, Wonju, Republic of Korea

**Keywords:** mental health, multicultural families, minorities, homogenous country, social network

## Abstract

We investigate the mental health of multicultural families (CFs) in South Korea, identify risk factors, and propose interventions to improve mental health. Adults over 19 years of age were analyzed using the Community Health Survey 2019 in South Korea, consisting of 228,952 individuals including 3,524 from multi-CFs. We employed chi-squared tests and multiple logistic regression to compare mental health between multi- and mono-CFs, exploring the influence of various factors. Multi-CFs had significantly higher levels of stress recognition (*P*-value = 0.010) and experiences of extreme sadness or despair (*P*-value = 0.002) than mono-CFs. In multi-CFs, younger group, households with lower income and people with unhealthy behaviors regarding walking or sleeping were at risk of mental health. Socially isolated families, relative to the families participating in active social gatherings, had about a 1.36 times higher risk of stress, 2 times higher experiences of extreme sadness or despair and 5.32 times higher depressive symptoms. Multi-CFs are vulnerable to mental health problems, and even within multi-CFs, groups with relatively low socioeconomic status should be prioritized since problems are more significant among them. Activated social networks can help multi-CFs integrate into society and promote mental health.

## Graphical abstract

**Figure:**
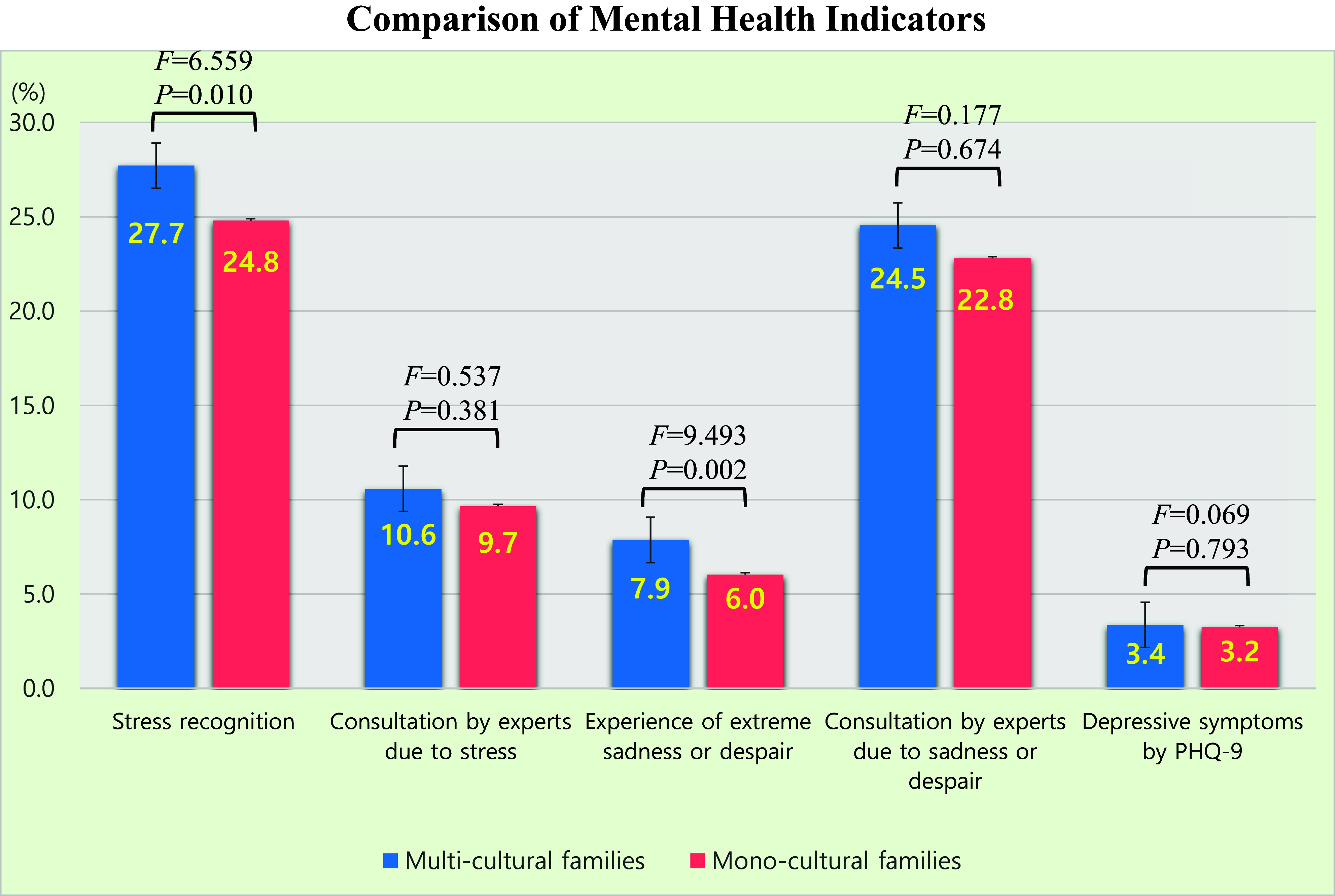

## Impact statement

This study provides crucial insights into the mental health status and risk factors faced by multicultural families (multi-CFs) in South Korea, a racially homogeneous country where such families face unique challenges. The findings reveal that multi-CFs experience significantly higher levels of stress, extreme sadness and despair compared to monocultural families (mono-CFs). These mental health disparities are exacerbated by precarious socioeconomic environments, unhealthy health behaviors and social isolation. Specifically, economic disadvantages, unstable marital statuses and risky health behaviors such as smoking and insufficient physical activity further worsen mental health outcomes among multi-CFs. The study highlights the urgent need for tailored intervention strategies that address these specific challenges. Key recommendations include promoting physical activity, enhancing social support networks and removing cultural and socioeconomic barriers to accessing mental health care. Furthermore, the household-level nature of mental health vulnerabilities in multi-CFs suggests that interventions should target entire families rather than focusing solely on individual members. This holistic approach is essential for breaking the cycle of disadvantage and improving overall well-being within these families. Despite certain limitations, such as the exclusion of individuals with poor Korean language skills and children under 19, this study contributes valuable knowledge to the understanding of mental health disparities in multi-CFs within a unique sociocultural context. The results underscore the importance of developing culturally sensitive, family-centered mental health interventions to address the specific needs of multi-CFs in South Korea and other similarly homogeneous societies.

## Introduction

Amid globalization, multicultural families (CFs) are increasing and becoming common worldwide. The US is a representative multicultural society that has experienced a rapid increase in immigrants, who comprised 13.9% of the total U.S. population in 2022 (Migration Policy Institute, 2024). As of January 2023, 6.1% of people living in EU countries are non-EU citizens (Eurostat, 2024). Even countries that have historically had monocultural populations are no exception.

### Social context of South Korea

South Korea has long emphasized ethnic and genealogical homogeneity, but since the late 20th century, the number of multi-CFs have risen owing to an influx of foreign workers, increased immigration through international marriages and globalization (Kim, 2007). As of 2022, there are approximately 399,000 multi-CFs in South Korea, accounting for 1.83% of the total number of households (Statistics Korea, 2023). As of 2022, school-aged children from multi-CFs number approximately 169,000. Although the total student population in Korea declined by 7.9% during the 2017–2022 period due to the declining fertility rate, the number of students from multi-CFs increased by 54.2% during the same period (Ministry of Gender Equality and Family, 2023a).

However, multi-CFs in South Korea are distinct from their counterparts in immigrant-based countries with a highly heterogeneous population and multiculturalist societies, such as the US and Canada. The majority are formed through marriages between female migrants and Korean men, with the gender ratio of marriage migrants being 78.6% female and 21.4% male. Additionally, marriage migrants are primarily from other Asian countries, particularly China (51.3%), Vietnam (21.8%) and the Philippines (5.4%), with these countries accounting for about 82% of all countries of origin (Ministry of Gender Equality and Family, 2023b). In terms of the Korean sociocultural context, these families face significant challenges in acculturating. An international survey on national identity indicates that 71.2% of South Koreans consider ancestry an essential criterion for being a genuine country member, strikingly higher than the percentages in Switzerland (35.5%) and the US (42.2%) (ISSP Research Group, 2015). Furthermore, 69.8% of marriage migrants and naturalized citizens have reported facing discrimination in their workplace solely for being a foreigner (Ministry of Gender Equality and Family, 2022).

### High risk of mental illness on migrants and ethnic minorities

Thus, mental health disorders of multi-CFs and migrants are a major public health concern. Owing to the cultural milieu and socioeconomic adversities they face, ethnic minorities are at a heightened risk of a range of mental illnesses such as stress, anxiety and depression, and exhibit a higher incidence of suicide and admission for suicide or self-harm (Karlsen and Nazroo, 2002; Ngwena, 2014; Wyatt et al., 2015; Forte et al., 2018). Indeed, rates of psychosis among immigrants are much higher than those among native populations, with an incidence rate ratio more than three times higher (Castillejos et al., 2018). For displaced populations such as refugees, psychiatric disorders, including post-traumatic stress disorder (PTSD), anxiety and depression, are more prevalent (Blackmore et al., 2020; Bedaso and Duko, 2022). Additionally, marriage migrants, who are already encumbered by their identity as an ethnic minority, experience gender vulnerability and family conflicts, which collectively engender a higher risk of social exclusion, depression and suicide (Wyatt et al., 2015; Kim, 2018). Even offspring of immigrants experience higher risk of depression compared to the host population, resulting from their adverse socioeconomic status (SES) and perceived ethnic discrimination (Stronks et al., 2020).

Despite the high need for mental health care among migrants and ethnic minorities, they have poor access to and limited use of mental health services. Lack of trust in healthcare providers, cultural beliefs, concerns about potential stigmatization, lack of insurance, language barriers and challenges in seeking help may be influencing factors (Heredia Montesinos, 2015; Derr, 2016; Mohammadifirouzeh et al., 2023). Such delayed help-seeking often results in treatment delays and subsequently affects treatment outcomes in psychotic disorders (Albert et al., 2017).

### Study objectives and necessity

In South Korea, multi-CFs refer to households with at least one member who is an ethnic minority. Marriage migrants can become naturalized Korean citizens when they fulfill the stipulated requirements, which includes duration of stay in the country. The Korean government enacted the Multicultural Families Support Act in 2008 and has operated 230 centers for multi-CFs nationwide. However, the government support program mainly focuses on Korean language education for marriage migrants and basic academic skills for their children. The Korean public has consistently reported a low level of receptiveness to multiculturalism (Ministry of Gender Equality and Family, 2023a). Furthermore, in terms of the national identity, Korean people tend to place more value on ancestry over country of birth or naturalization (ISSP Research Group, 2015), raising concerns about perpetuation and generational transmission of adverse social experiences. Moreover, research on mental health and racial minorities has mainly been conducted in multiethnic countries such as the US, and thus may not be applicable to mono-ethnic or monoracial countries such as South Korea. Accordingly, the current study may serve as a prime example of research for mono-ethnic countries.

This study aims to compare stress recognition, the experience of sadness or despair and depressive symptom prevalence between mono- and multi-CFs. In addition, we identify the risk factors of these indicators in multi-CFs. We subsequently provide countermeasures for improving mental health equity in multi-CFs.

### Theoretical background

As the notion of an immigrant encompasses a complex construal of distinct nationality, culture, race and ethnicity, contextual factors affect their mental health, ranging from personal abilities (e.g., language competency), family, neighborhood and social position to social experiences (Alegría et al., 2017). Immigration is a critical process that can be influenced by these social and institutional factors and, at the same time, lead to changes in each domain. Therefore, migrants’ health needs to be investigated through the lens of social determinants of health, incorporating behavioral, cultural and structural frameworks (Castañeda et al., 2015).

Additionally, the relation between social exclusion and mental health arises through the psychosocial stress process, indicating that cumulative exposures to social isolation and discrimination stress affect brain neurotransmitter systems and amplify psychotic and affective disorders (Brandt et al., 2022). It is worth noting that both income inequality and minority status among immigrants synergistically impair the chances for social participation, thereby increasing social exclusion (Heinz et al., 2020).

A great deal of previous studies have explored the effects of discrimination, acculturative stress, social exclusion and family conflict on migrants’ mental health. Migrants undergo a process of acculturation, wherein they are integrated into a culture by accepting the values, beliefs and attitudes of the culture, and the cultural conflict and acculturative stress they experience serve as major barriers to their mental health (Hovey and Magaña, 2003; Nazroo, 2003; Heredia Montesinos, 2015; Wyatt et al., 2015; Forte et al., 2018). Language proficiency is associated with mental disorders in migrants, including mood, stress, anxiety and psychotic disorders (Montemitro et al., 2021). Particularly in South Korea, Korean language proficiency in multi-CFs has been linked to the risk of social exclusion (Kim, 2018). Moreover, migrants’ experiences of interpersonal racism, institutional discrimination and household socioeconomic disadvantage have been identified as crucial contributors to the level of their depression, suicide risk and psychosis (Karlsen and Nazroo, 2002; Nazroo, 2003; Juang and Cookston, 2009; Stein et al., 2014; Heredia Montesinos, 2015; Wang et al., 2021). In terms of social status, migrants who experience a downward shift in social status as a result of their relocation to another country compared to that in their home country are at a greater risk of developing common mental disorders (Das-Munshi et al., 2012). Beyond economic capital, social capital can have profound implications for migrants’ mental health. Social exclusion, social isolation, disruption of social networks, lack of social support and separation from family, all following migration, increase the risk of depressive disorders, suicidal acts and suicide among migrants (Hagaman et al., 2016; Alegría et al., 2017). Meanwhile, immigrants’ cultural resilience serves a protective role in preventing the onset of depression, and their high levels of religiosity and faith sometimes act as coping strategies (Cervantes et al., 2019; Lusk et al., 2021).

Particularly concerning familial factors, they can act as both stressors and buffers for migrants’ mental health. Familial and interpersonal problems, such as domestic violence and pressures from the community, were major risk factors for suicide among migrants and ethnic minorities (Heredia Montesinos, 2015). In the context of multi-CFs in Korea, marriage migrant mothers’ experiences of discrimination influence maternal depression, which in turn affects their parenting behavior and their children’s psychological adjustment (Chung and Lim, 2016). Meanwhile, family reunification and familism during post-migration tend to decrease the risk of depressive symptoms for immigrant parents (Ornelas and Perreira, 2011). Parental support attenuates migrant children’s issues such as identity confusion and psychological well-being (Walsh et al., 2012). Similarly, adolescents from multi-CFs in Korea benefit from support from family, friends and teachers, which facilitates higher acculturation. As their acculturation progresses toward social integration, they report greater life satisfaction (Yoo, 2021).

## Methods

### Data and participants

We used data from the Community Health Survey (CHS) of Korea. CHS surveys 900 adults over 19 years of Korean citizenship by district unit by Korea Disease Control Prevention and Control Agency (KDCA) and local governments since 2008, surveying approximately 230,000 people yearly. To produce representative statistics, CHS conducts household unit surveys through a multistage stratified sample design. In addition, the survey on multi-CFs, which is the main research group of our study, has been conducted every 3 years from 2019. Therefore, the study subjects were 228,952 adults aged 19 and older in 2019, of which 3,524 responded that they were from multicultural households. CHS classifies a multicultural household a family comprised of people from different nationalities, races and cultural backgrounds. A family is also classified as multi-CF if it includes a person who emigrated from a foreign country even though they acquired Korean nationality, or if the respondent was born in Korea but at least one parent was born in a foreign country and answered that they consider themselves to be multicultural.

### Variables

To compare the level of mental health in mono- and multi-CFs, we selected stress recognition, experience of extreme sadness or despair and depressive symptoms as the primary dependent variables. These variables were chosen based on their relevance to mental health as indicated in previous research. For instance, stress recognition has been consistently linked to mental health outcomes, where higher levels of perceived stress are associated with increased risks of anxiety and depression (Cohen et al., 1983; Lazarus, 1984). Similarly, experiences of extreme sadness or despair are critical indicators of mental health status, often predictive of depressive disorders (Kessler et al., 2003). Depressive symptoms themselves are widely recognized as core measures of mental health, reflecting both the presence and severity of depression (Kroenke et al., 2001). Therefore, selecting these variables allows for a comprehensive assessment of mental health, capturing a range of emotional and psychological states that are crucial for understanding overall well-being. Additionally, consultation with experts due to stress and consultation with experts due to sadness or despair were considered key mental health-related outcomes. We incorporated multiple social determinants of health for multi-CFs as independent variables. These range from demographics and health-promotion behaviors to household characteristics, SES and social capital, all of which are associated with potential discrimination, social experiences and coping strategies (Castañeda et al., 2015; Alegría et al., 2017). Household and socioeconomic factors include sex, age, region, beneficiary status for basic livelihood, monthly household income, education, occupation and marital status. Additionally, current smoking, monthly alcohol use, weekly walking and daily sleep duration were selected as health-related behaviors. Religious activities, social gatherings, leisure activities and charity activities were chosen as indicators of social capital (Table 1).

**Table 1.** Definition of variables

	Variables	Definition
Key factors	Stress recognition	Percentage of “extremely high” and “high” on a four-point response to “How high is your daily stress level?”
	Consultation by experts due to stress	Percentage of individuals with perceived stress who have sought counseling for stress at a health care facility, counseling facility or public health center.
	Experience of extreme sadness or despair	Percentage of “yes” to the question “Have you felt extremely sad or hopeless to the point of having disruptions to your daily life for two consecutive weeks or longer in the past 2 years?”
	Consultation by experts due to a sadness or despair	Among those answered “yes” to the above question, those who sought counseling at a health care facility, counseling facility or public health center.
	Depressive symptoms by PHQ–9	Score ≥10 on Patient Health Questionnaire–9
Social-economic factors	–	Sex, age, region, Basic livelihood beneficiary, education
	Household income per month	Unit: Million Korea Won, low: <1, lower middle: 1<=income<3, upper middle: 3<=income<6, high: 6<=income
	Occupation	Professional or administrative, general office worker, sales or service worker, agriculture or fisheries, skilled or simple labor (machinery, assembly workers), etc. (soldier, student, housewife, unemployed)
	Marital status	Spouse and live together, divorce, bereavement, separation, not married and single.
Health-related factors	Current smoker	Among those who have smoked at least 100 cigarettes in their lifetime, percentage who claimed to be a current smoker.
	Monthly alcohol use	People who consumed alcohol at least once a month on average in the past year.
	Weekly walking	People who walked 30 min or longer a day for 5 days or more in the past week.
	Daily sleep duration	Responses for different durations to the question “How much do you usually sleep each night?”
Social activities	Religious activities	People who answered “yes” to the question “Do you regularly participate in religious activities at least once a month?”
	Social gathering	People who answered “yes” to the question “Do you regularly participate in social activities at least once a month?”
	Leisure activities	People who answered “yes” to the question “Do you regularly participate in leisure activities at least once a month?”
	Charity activities	People who answered “yes” to the question “Do you regularly participate in charity activities at least once a month?”

### Statistical analysis

We performed descriptive statistics to compare the characteristics of mono- and multi-CFs. In addition, a chi-squared test compared the indicators related to stress and depressive symptoms in the two groups, and multiple logistic regression identified risk factors related to stress and depressive symptoms in multi-CFs. All analyses were performed by reflecting the multi-stratified cluster sampling with SAS for Windows 9.4 (SAS Institute Inc., NC, USA).

## Results

### Descriptive statistics

Of 228,952 respondents, 3,524 (1.9%) were from multi-CFs. The percentage of men (55.8%) was higher among multi-CFs, while the percentage of women (50.5%) was higher among mono-CFs. The most common age was <45 in both groups, and there were more people from urban regions than rural regions. The percentage of basic livelihood beneficiaries was higher among multi-CFs (3.3%) than mono-CFs (2.6%). By income level, upper middle class was the most common in both groups. In terms of education, high school (43.7%) was the most common among multi-CFs, while college or higher (42.2%) was that among mono-CFs. Skilled or simple labor (37.3%) and other (36.9%) were the most common occupations among multi-CFs and mono-CFs, respectively. The highest number of people lived with their spouse in both groups (multi-CFs: 82.8%; mono-CFs: 63.6%). The percentage of current smokers was higher among multi-CFs (multi-CFs: 26.9%; mono-CFs: 18.5%), while the percentages of people engaging in monthly alcohol drinking (multi-CFs: 51.8%; mono-CFs: 57.0%) and weekly walking were higher among mono-CFs (multi-CFs: 46.9%; mono-CFs: 50.3%). The most common daily sleep duration was 6–8 h in both groups. Finally, proxies of social activities, namely religious activities, social gathering, leisure activities and charity activities were higher in mono-CFs than multi-CFs (Table 2).

**Table 2.** Descriptive analysis

		Multicultural families (*n*1)		Monocultural families (*n*2)	
		*N* (weighted no.)	Weighted %	*N* (weighted no.)	Weighted %
Sex (*n*1 = 3,524, *n*2 = 225,428)	Men	1,745 (359,434)	55.8	100,744 (20,955,473)	49.5
	Women	1,779 (284,502)	44.2	124,684 (21,414,630)	50.5
Age (*n*1 = 3,524, *n*2 = 225,428)	Age <45	1,325 (310,385)	48.2	64,910 (17,963,228)	42.4
	45≤ age <65	1,460 (268,324)	41.7	86,742 (16,268,473)	38.4
	65≤ age <75	373 (36,192)	5.6	38,713 (4,643,708)	10.9
	75≤ age	366 (29,035)	4.5	35,063 (3,494,692)	8.3
Region (*n*1 = 3,524, *n*2 = 225,428)	Urban	1,513 (472,777)	73.4	127,131 (34,524,865)	81.5
	Rural	2,011 (171,158)	26.6	98,297 (7,845,237)	18.5
Basic livelihood beneficiary (*n*1 = 3,521, *n*2 = 225,383)	No	3,382 (618,640)	96.1	216,462 (41,033,529)	96.9
	Ex	39 (4,023)	0.6	1,447 (208,969)	0.5
	Yes	100 (21,023)	3.3	7,474 (1,117,457)	2.6
Monthly household income (*n*1 = 3,524, *n*2 = 225,428)	Low	306 (42,003)	6.5	39,111 (4,603,979)	10.9
	Lower middle	1,231 (205,919)	32.0	67,958 (10,077,498)	23.8
	Upper middle	1,654 (320,569)	49.8	81,968 (18,050,518)	42.6
	High	333 (75,444)	11.7	36,391 (9,638,107)	22.7
Education (*n*1 = 3,520, *n*2 = 225,206)	No education	325 (23,900)	3.7	22,701 (1,808,148)	4.3
	Elementary	494 (54,038)	8.4	34,349 (3,565,135)	8.4
	Middle	560 (95,751)	14.9	24,930 (3,422,737)	8.1
	High	1,419 (281,364)	43.7	73,938 (15,657,849)	37.0
	Above college	722 (188,381)	29.3	69,288 (17,878,391)	42.2
Occupation (*n*1 = 3,518, *n*2 = 225,120)	General office worker	136 (40,029)	6.2	19,541 (5,152,564)	12.2
	Professional or administrative	191 (55,917)	8.7	22,988 (6,083,333)	14.4
	Sales or service worker	425 (91,523)	14.2	29,641 (5,979,967)	14.1
	Agriculture or fisheries	578 (39,983)	6.2	24,765 (1,340,271)	3.2
	Skilled or simple labors	1,190 (239,642)	37.3	41,646 (8,117,606)	19.2
	Etc.	998 (176,040)	27.4	86,539 (15,621,491)	36.9
Marital status (*n*1 = 3,524, *n*2 = 225,212)	Spouse and live together	2,818 (533,221)	82.8	149,179 (26,920,002)	63.6
	Divorce	99 (21,155)	3.3	9,360 (1,751,648)	4.1
	Bereavement	383 (36,013)	5.6	28,350 (3,023,637)	7.1
	Separation	54 (9,029)	1.4	3,154 (498,841)	1.2
	Single	170 (44,519)	6.9	35,169 (10,133,123)	23.9
Current smoker (*n*1 = 3,524, *n*2 = 225,415)	Yes	827 (172,952)	26.9	36,407 (7,829,757)	18.5
Monthly alcohol use (*n*1 = 3,523, *n*2 = 225,398)	Yes	1,588 (333,550)	51.8	111,133 (24,139,919)	57.0
Weekly walking (*n*1 = 3,524, *n*2 = 206,717)	Yes	1,276 (277,782)	46.9	90,862 (19,413,378)	50.3
Daily sleep duration (*n*1 = 3,523, *n*2 = 225,371)	<6	540 (89,776)	13.9	40,007 (6,914,795)	16.3
	6 ≤ h <8	1,980 (381,991)	59.3	176,506 (33,998,902)	80.3
	≥8 h	1,003 (172,027)	26.7	8,858 (1,448,933)	3.4
Religious activities (*n*1 = 3,522, *n*2 = 225,411)	Yes	757 (126,700)	19.7	61,460 (11,254,698)	26.6
Social gathering (*n*1 = 3,522, *n*2 = 225,411)	Yes	1,538 (255,063)	39.6	123,613 (22,258,347)	52.5
Leisure activities (*n*1 = 3,522, *n*2 = 225,411)	Yes	582 (142,535)	22.1	63,088 (14,311,211)	33.8
Charity activities (*n*1 = 3,522, *n*2 = 225,411)	Yes	206 (33,632)	5.2	18,612 (3,365,241)	7.9

### Stress recognition, experience of extreme sadness or despair and depressive symptoms between multicultural and monocultural families

**Figure 1.**
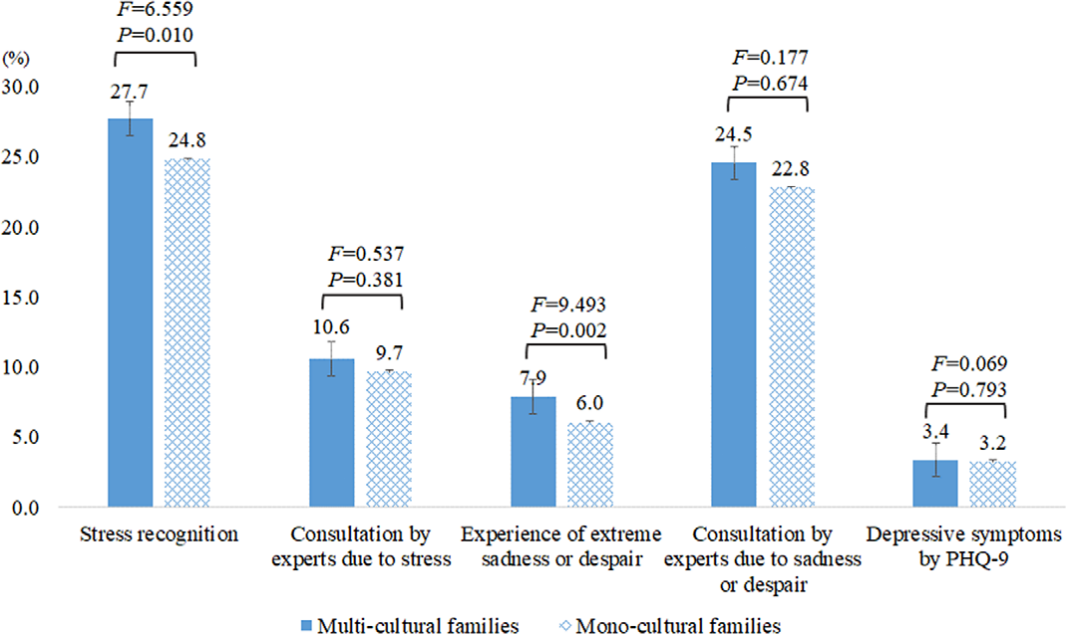
Comparison of stress recognition, sadness or despair and depressive symptoms of multicultural and monocultural families.

Stress recognition was significantly higher among multi-CFs (27.7%) than mono-CFs (24.8%) (*F* = 6.559, *P* = 0.010), while the resulting rate of counseling did not differ significantly between the two groups. The percentage of individuals who have experienced extreme sadness or despair was significantly higher among multi-CFs (7.9%) than mono-CFs (6.0%), but the resulting rate of counseling did not differ significantly between the two groups. Finally, the prevalence of depressive symptoms was higher in multi-CFs (3.4%) than mono-CFs (2.9%) but not to a significant extent (Figure 1).

### Risk factors in stress recognition, experience of extreme sadness or despair and depressive symptoms in multicultural families

Stress recognition did not differ between genders. The odds for stress recognition were higher (OR = 2.93) in the ≥75 years group than the <45 years group, in the urban region (OR = 1.14) than rural region, and among current beneficiaries (OR = 1.93) than non-beneficiaries. Stress recognition did not differ significantly according to household income. Regarding those with a college degree or higher, the odds for stress recognition were higher among those without education (OR = 1.72) but lower among those with middle school (OR = 0.66) and high school education (OR = 0.84). The odds for stress recognition among office workers were lower among those in agriculture or fisheries (OR = 0.59). In terms of marital status, the odds were higher among those who are divorced (OR = 1.82) or separated (OR = 1.50) than those who live with their spouse. The odds were higher among current smokers (OR = 1.19) and those who do not walk weekly (OR = 1.25). Moreover, the odds were higher among those who sleep fewer than 6 h a day (OR = 3.48) than those who sleep 6–8 h a day, higher among those who do not have social gatherings (OR = 1.36) and lower among those who do not engage in leisure activities (OR = 0.84).

The odds for sadness or despair were higher among men than women (OR = 1.57) and highest among the 45–64 years group compared to the ≥75 years group (OR: 4.00). However, depressive symptoms did not differ significantly according to sex and age. By region, the odds for depressive symptoms were higher in the urban region (OR = 1.48). The odds for sadness or despair (OR = 1.50) and depressive symptoms (OR = 4.75) were higher among the current basic livelihood beneficiary group than the non-beneficiary group, while the odds were 2.51 and 5.60 in the ex-beneficiary group compared to the non-beneficiary group, respectively. In terms of household income, the odds for sadness or despair (OR = 2.50) and depressive symptoms (OR = 3.22) were higher in the low-income group compared to the high-income group. Regarding those with a college degree or higher, the odds for sadness or despair were higher among the no-education (OR = 1.37) and elementary groups (OR = 2.19), while those for depressive symptoms were lower in the middle school group (OR = 0.38). With reference to office workers, with the exception of the professional or administrative group having no significant differences, all other occupations had significantly lower odds for both sadness or despair and depressive symptoms. In terms of marital status, the odds for sadness or despair were higher in the bereaved group (OR = 1.64) than the group living with their spouse. Further, for the group living with their spouse, the odds for depressive symptoms were higher in the separated group (OR = 2.06) but lower in the divorced group (OR = 0.2). There were no significant differences according to current smoking and drinking status. In terms of weekly walking, the odds for sadness or despair and depressive symptoms were both higher in the “no” group. For the group who sleep 6–8 h a day, the <6 h group had higher odds for sadness or despair (OR = 2.24) and depressive symptoms (OR = 6.87). The odds for depressive symptoms were higher (OR = 1.75) in the no religious activities group, and the odds for sadness/despair (OR = 2.00) and depressive symptoms (OR = 5.32) were higher in the social gathering group (Table 3).

**Table 3.** Results of multiple logistic analysis of stress, extreme sadness or hopelessness and depressive symptoms in multicultural families

Variables	Categories	Stress recognition	Extreme sadness or despair	Depressive symptoms (PHQ ≥ 10)
		OR (95% CI)	OR (95% CI)	OR (95% CI)
Sex (ref.: men)	Women	1.12 (0.96–1.30)	1.57 (1.08–2.26)	0.92 (0.48–1.75)
Age (ref.: 75≤ age)	Age <45	2.93 (2.14–4.01)	3.34 (1.31–8.51)	0.73 (0.26–2.03)
	45≤ age <65	2.06 (1.52–2.80)	4.00 (1.62–9.88)	0.84 (0.35–2.02)
	65≤ age <75	1.24 (0.91–1.68)	2.45 (1.12–5.37)	1.01 (0.48–2.10)
Region (ref.: rural)	Urban	1.14 (1.01–1.28)	0.91 (0.74–1.12)	1.48 (1.06–2.05)
Basic livelihood beneficiary (ref.: no)	Current beneficiary	1.93 (1.38–2.69)	1.50 (0.83–2.72)	4.75 (2.50–9.02)
	Ex-beneficiary	0.99 (0.49–2.01)	2.51 (1.53–4.11)	5.60 (3.52–8.92)
Household income per month (ref.: high)	Low	0.91 (0.65–1.26)	2.59 (1.37–4.89)	3.22 (1.75–5.92)
	Lower middle	1.00 (0.79–1.26)	1.63 (1.23–2.16)	2.16 (1.38–3.38)
	Upper middle	0.89 (0.72–1.09)	0.84 (0.63–1.12)	0.61 (0.31–1.20)
Education (ref.: above college)	No-education	1.72 (1.32–2.25)	1.37 (0.93–2.01)	1.03 (0.61–1.72)
	Elementary	1.10 (0.88–1.36)	2.19 (1.46–3.30)	0.81 (0.44–1.48)
	Middle	0.66 (0.53–0.83)	1.06 (0.74–1.51)	0.38 (0.23–0.65)
	High	0.84 (0.72–0.97)	1.12 (0.80–1.56)	1.18 (0.68–2.02)
Occupation (ref.: office worker)	Professional or administrative	0.92 (0.68–1.23)	0.82 (0.47–1.42)	0.34 (0.05–2.43)
	Sales or service worker	1.08 (0.80–1.45)	0.50 (0.29–0.87)	0.29 (0.12–0.73)
	Agriculture or fisheries	0.59 (0.45–0.79)	0.22 (0.13–0.36)	0.05 (0.02–0.16)
	Skilled or simple labors	0.84 (0.66–1.07)	0.39 (0.25–0.62)	0.20 (0.06–0.64)
	Etc.	0.79 (0.62–1.02)	0.51 (0.33–0.79)	0.41 (0.17–1.00)
Marital status (ref.: spouse and live together)	Divorce	1.82 (1.29–2.56)	1.58 (0.95–2.62)	0.20 (0.11–0.36)
	Bereavement	0.80 (0.61–1.05)	1.64 (1.06–2.53)	1.70 (0.65–4.47)
	Separation	1.50 (1.03–2.19)	1.64 (0.80–3.37)	2.06 (1.05–4.02)
	Single	0.84 (0.66–1.08)	0.99 (0.48–2.06)	0.70 (0.39–1.26)
Current smoker (ref.: no)	Current smoker	1.19 (1.02–1.39)	0.79 (0.50–1.23)	1.23 (0.61–2.47)
Monthly alcohol use (ref.: no)	Monthly drinker	0.91 (0.79–1.04)	0.94 (0.71–1.25)	0.69 (0.37–1.27)
Weekly walking (ref.: yes)	No	1.25 (1.11–1.41)	1.31 (1.02–1.68)	1.82 (1.13–2.93)
Daily sleep duration (ref.: 6≤ h <8)	<6 h	3.48 (2.60–4.65)	2.24 (1.71–2.92)	6.87 (4.28–11.02)
	≥8 h	1.56 (1.22–2.01)	1.66 (1.21–2.28)	1.70 (0.97–2.97)
Religious activities (ref.: yes)	No	1.04 (0.93–1.17)	1.06 (0.86–1.32)	1.75 (1.16–2.63)
Social gathering (ref.: yes)	No	1.36 (1.20–1.53)	2.00 (1.57–2.54)	5.32 (2.93–9.69)
Leisure activities (ref.: yes)	No	0.84 (0.71–0.98)	0.79 (0.51–1.23)	0.92 (0.31–2.73)
Charity activities (ref.: yes)	No	1.29 (0.99–1.69)	0.90 (0.61–1.32)	1.09 (0.79–1.49)

## Discussion

Multi-CFs in South Korea are vulnerable groups with higher levels of stress and experiences of extreme sadness or despair relative to mono-CFs. Additionally, multi-CFs are likely to be positioned in risky environments regarding lower SES, a high prevalence of smoking, a shortage of walking practice, abnormal sleeping times and social isolation, all of which are directly linked to a decrease in mental health. Interventions must promote health activities and help families from different ethnic backgrounds get involved in their communities.

Stress recognition and extreme sadness or despair among multi-CFs were significantly higher than mono-CFs, but there was no difference in the level of depressive symptoms and specialist consultation rate. This result contradicts previous studies and reveals the mental health characteristics of migrant women in multi-CFs in the social context of South Korea. According to review papers comparing mental health levels of immigrants and nonimmigrants, the results for suicidal behavior and death by suicide were inconsistent, with no difference or better than nonimmigrants in some cases (Forte et al., 2018).

### Vulnerabilities in women or younger people from multi-CFs regarding mental health

In our analysis of mental health risks among multi-CFs, stress and sadness or despair were more prevalent in women compared to men and in younger adults compared to older age. Previous studies have also discussed the impact of sociodemographic factors on migrants’ mental health. Migrants’ country of origin was significantly associated with suicide rate (Bhui and McKenzie, 2008; Wong et al., 2014; Forte et al., 2018), and younger female immigrants (Montesinos et al., 2013); in particular, young female immigrants from South Asia have been identified as a major vulnerable group for mental health (Spallek et al., 2015). In a study of South Asian (SA) immigrants in the US and UK, SA women and SA youth exhibited poor treatment-seeking patterns due to low trust in practitioners and adherence to collectivist family norms. Experiences of domestic violence, financial pressure and low self-esteem further exacerbate gender and age disparities in depression. For SA youth, poor acculturation and discrimination increase stress (Karasz et al., 2019). In the Korean context, a study sheds light on age and time concerns among Asian female marriage migrants in Korea. It reported better acculturation with longer residence in Korea, while individuals aged 30–45, compared to older individuals, experienced significantly higher levels of acculturative stress, even after adjusting for other demographics (Kim et al., 2022). These recurring tendencies underscore the importance of cultural norms and duration of residence related to acculturation, rather than biological reasons.

### Impact of economic disadvantages and unstable marital status on multi-CFs

Regarding SES factors, economic disadvantages, such as families receiving benefits or having low household income, were significant risk factors for extreme sadness or despair and depressive symptoms in this study. Interestingly, middle/high school graduates showed some protective effects on mental health compared to college graduates. Office workers had a higher risk, whereas other occupations were associated with lower risks of mental illness, contrasting with previous findings. According to a WHO survey, higher education levels in high-income countries improve treatment compliance for severe or moderate mental disorders (Evans-Lacko et al., 2018). In the UK, migrants from ethnic minorities, with low household income, and not economically active had more than five times the odds ratio of common mental disorders compared to nonmigrant white British individuals with professional occupations, high income and education (Goodwin et al., 2018). Our results highlight the greater importance of economic concerns for the mental health of multi-CFs compared to other aspects of SES, such as occupation or educational achievement.

The impact of marital status on mental health was inconsistent for different parameters. In general, unstable marital status adversely affected mental health of multi-CFs, but the risk of depressive symptoms was significantly lower among the divorced group compared to the living with spouse group. This may be because spousal conflicts among non-divorced families may cause depressive symptoms. A total of 21.2% of marriage migrants in South Korea have experienced discrimination from family members because of their foreign origin (Ministry of Gender Equality and Family, 2022), highlighting the serious level of discord within families. Indeed, the primary concern for marriage migrants in Asia is the relationship among extended family members, unlike in Western countries, which are predominantly interested in race and ethnic integration (Yeung and Mu, 2020). In a comparison of intra-Asian countries, Vietnamese marriage migrants in South Korea, compared to those in Taiwan, experience more rigid gender expectations related to the family role as daughters-in-law. These gender systems and cultural norms subject female migrants to gender-based and ethnic discrimination (Chang, 2020).

### Need for enhancing health behaviors and social participation

Regarding health behaviors, multi-CFs exhibited higher rates of smoking, lower levels of physical activity (walking) and unhealthy sleep durations compared to Korean families, and these health risk behaviors have emerged as significant factors influencing their mental health. Based on a study in South Korea, Park et al., 2018 reported that, compared to those from mono-CFs, children from multi-CFs display higher rates of alcohol use, smoking and drug use, alongside an increased prevalence of psychological problems such as depressed mood, suicidal ideation and attempts (Park et al., 2018). We can also consider the possibility of somatization. Untreated mental illnesses are often presented as somatic rather than mental symptoms, manifesting as physical issues like sleep abnormalities, bodily pains or gastrointestinal problems (Karasz et al., 2019). In essence, health behaviors in multi-CFs in South Korea are notably suboptimal, and the amelioration of these health behaviors is crucial for enhancing both physical and mental health.

Social participation has been reported to have positive effects on mental health. The main channels through which migrants receive social support and seek help regarding their mental health are family, friends or religious leaders, and social support from these individuals lowers immigration stress (Derr, 2016; Sanchez et al., 2019). Furthermore, social capital mediates the association between immigrant status (i.e., time since immigration, asylum status) and psychological distress (Johnson et al., 2017), and immigrants with rich social networks in Korean society are at a low risk of social exclusion (Kim, 2018). Moreover, social capital in multi-CFs is important because maternal social capital leads to children’s physical growth and health. Social networks function as crucial information channels, enabling mothers to obtain essential knowledge about parenting attitudes, nutrition and child-rearing practices that can profoundly impact their children’s nutritional status (De Silva and Harpham, 2007). We observed a correlation between increased leisure activity participation and higher stress recognition. Despite this association, due to the limitations of the cross-sectional design in establishing causal relationships, it can be interpreted that people with high stress levels are more likely to engage in leisure activities to manage stress.

### Implication and limitations

In summary, we observed social deprivations among multi-CFs in Korean society, accompanied by elevated levels of stress recognition and experiences of extreme sadness or despair compared to mono-CFs. Considering their lower SES and social isolation, as well as its strong association with mental distress in this study, the mental health of multi-CFs could worsen over time if there are no adequate interventions. Intervention strategies should be prioritized toward mitigating economic hardship, improving their health behaviors and increasing social participation, thereby helping them break the cycle of disadvantages. These goals can be achieved efficiently by promoting physical activity and expanding opportunities to participate in more social gatherings. Finally, since the scope of analysis was limited to members of multi-CFs as opposed to individual immigrants, our results depict the mental health status in household units. In the same context, mental health vulnerability is likely to be linked to the household characteristics of multi-CFs rather than individual issues, so we argue that household-level interventions, and not individual-level interventions, are needed for immigrant families.

This study has several limitations. First, in the case of nationality acquired through marriage, those with poor Korean communication skills were excluded from the survey. In the case of marriage migrants, the proportion of women is much higher (Ministry of Gender Equality and Family, 2023b). Therefore, this result may be an underestimation of the mental health outcomes of immigrant and ethnic minorities (mainly women). Second, the cross-sectional CHS data we used has limitations in investigating overall life-course perspectives. It does not cover children under 19 and is not suitable for further subdividing multi-CFs by age group due to the small sample size. Given that risk factors can vary by age and that childhood experiences of youth in multi-CFs can impact their adulthood mental health, future studies need to consider age segments or generational effects. Accumulating data over several years can enable the acquisition of a sufficient sample size and more precise study targets. Third, in our study, responses related to stress or anxiety were measured using a Likert scale or ‘yes or no’ format. However, using more sophisticated tools such as the Perceived Stress Scale (PSS) or the Depression Anxiety Stress Scales (DASS) could allow for a more robust and in-depth analysis. Lastly, the absence of information on immigrants’ countries of origin made it impossible to discriminate ethnic differences in detail or reflect them in the investigation. However, despite these limitations, this study is meaningful in that it identified the mental health status and risk factors of multi-CFs in the sociocultural context of a homogeneous country, which is different from the existing US and European countries.

## Conclusions

Multi-CFs exhibited higher levels of recognition of stress and experience of extreme sadness or despair than mono-CFs. Even within multi-CFs, since the problems of groups with relatively low SES were greater, this group should be considered as the priority group for intervention. This group is placed in a socially, economically and culturally vulnerable environment, and low SES and poor social activities and networks increase stress and despair. Social isolation should be improved, and social integration and health equity should be achieved by supporting multi-CFs’ diverse social participation. This study is meaningful in that it derived findings about the mental health problems experienced by multi-CFs in a homogeneous country and expands the variation of the sociocultural context.

## Data Availability

The data are available from KDCA website. If you need the processed data, please contact the corresponding author to request the data.

## References

[r1] Albert N, Melau M, Jensen H, Hastrup LH, Hjorthøj C and Nordentoft M (2017) The effect of duration of untreated psychosis and treatment delay on the outcomes of prolonged early intervention in psychotic disorders. NPJ Schizophrenia 3(1), 34. 10.1038/s41537-017-0034-4. 28951544 PMC5615058

[r2] Alegría M, Álvarez K and Dimarzio K (2017) Immigration and mental health. Current Epidemiology Reports 4(2), 145–155. 10.1007/s40471-017-0111-2. 29805955 PMC5966037

[r3] Bedaso A and Duko B (2022) Epidemiology of depression among displaced people: A systematic review and meta-analysis. Psychiatry Research 311, 114493. 10.1016/j.psychres.2022.114493. 35316692

[r4] Bhui KS and McKenzie K (2008) Rates and risk factors by ethnic group for suicides within a year of contact with mental health services in England and Wales. Psychiatric Services 59(4), 414–420. 10.1176/ps.2008.59.4.414. 18378841

[r5] Blackmore R, Boyle JA, Fazel M, Ranasinha S, Gray KM, Fitzgerald G, Misso M and Gibson-Helm M (2020) The prevalence of mental illness in refugees and asylum seekers: A systematic review and meta-analysis. PLoS Medicine 17(9), e1003337. 10.1371/journal.pmed.1003337. 32956381 PMC7505461

[r6] Brandt L, Liu S, Heim C and Heinz A (2022) The effects of social isolation stress and discrimination on mental health. Translational Psychiatry 12(1), 398. 10.1038/s41398-022-02178-4. 36130935 PMC9490697

[r7] Castañeda H, Holmes SM, Madrigal DS, Young M-ED, Beyeler N and Quesada J (2015) Immigration as a social determinant of health. Annual Review of Public Health 36(1), 375–392. 10.1146/annurev-publhealth-032013-182419. 25494053

[r8] Castillejos MC, Martín-Pérez C and Moreno-Küstner B (2018) A systematic review and meta-analysis of the incidence of psychotic disorders: The distribution of rates and the influence of gender, urbanicity, immigration and socio-economic level. Psychological Medicine 48(13), 2101–2115. 10.1017/S0033291718000235. 29467052

[r9] Cervantes RC, Gattamorta KA and Berger-Cardoso J (2019) Examining difference in immigration stress, acculturation stress and mental health outcomes in six Hispanic/Latino nativity and regional groups. Journal of Immigrant and Minority Health 21(1), 14–20. 10.1007/s10903-018-0714-9. 29488133

[r10] Chang H-C (2020) Do gender systems in the origin and destination societies affect immigrant integration? Vietnamese marriage migrants in Taiwan and South Korea. Journal of Ethnic and Migration Studies 46(14), 2937–2955. 10.1080/1369183X.2019.1585014.

[r11] Chung GH and Lim JY (2016) Marriage immigrant mothers’ experience of perceived discrimination, maternal depression, parenting behaviors, and adolescent psychological adjustment among multicultural families in South Korea. Journal of Child and Family Studies 25(9), 2894–2903. 10.1007/s10826-016-0445-2.

[r12] Cohen S, Kamarck T and Mermelstein R (1983) A global measure of perceived stress. Journal of Health and Social Behavior 24(4), 385–396. 10.2307/2136404. 6668417

[r13] Das-Munshi J, Leavey G, Stansfeld S and Prince M (2012) Migration, social mobility and common mental disorders: Critical review of the literature and meta-analysis. Ethnicity & Health 17(1–2), 17–53. 10.1080/13557858.2011.632816. 22074468

[r14] De Silva MJ and Harpham T (2007) Maternal social capital and child nutritional status in four developing countries. Health & Place 13(2), 341–355. 10.1016/j.healthplace.2006.02.005. 16621665

[r15] Derr AS (2016) Mental health service use among immigrants in the United States: A systematic review. Psychiatric Services 67(3), 265–274. 10.1176/appi.ps.201500004. 26695493 PMC5122453

[r16] Eurostat (2024) Statistics Explained: Migration and Migrant Population Statistics. Available at https://ec.europa.eu/eurostat/statistics-explained/index.php?title=Migration_and_migrant_population_statistics (accessed 24 June 2024).

[r17] Evans-Lacko S, Aguilar-Gaxiola S, Al-Hamzawi A, Alonso J, Benjet C, Bruffaerts R, Chiu W-T, Florescu S, de Girolamo G and Gureje O (2018) Socio-economic variations in the mental health treatment gap for people with anxiety, mood, and substance use disorders: Results from the WHO world mental health (WMH) surveys. Psychological Medicine 48(9), 1560–1571. 10.1017/S0033291717003336. 29173244 PMC6878971

[r18] Forte A, Trobia F, Gualtieri F, Lamis DA, Cardamone G, Giallonardo V, Fiorillo A, Girardi P and Pompili M (2018) Suicide risk among immigrants and ethnic minorities: A literature overview. International Journal of Environmental Research and Public Health 15(7), 1438. 10.3390/ijerph15071438. 29986547 PMC6068754

[r19] Goodwin L, Gazard B, Aschan L, Maccrimmon S, Hotopf M and Hatch SL (2018) Taking an intersectional approach to define latent classes of socioeconomic status, ethnicity and migration status for psychiatric epidemiological research. Epidemiology and Psychiatric Sciences 27(6), 589–600. 10.1017/s2045796017000142. 28390448 PMC6998994

[r20] Hagaman AK, Sivilli TI, Ao T, Blanton C, Ellis H, Lopes Cardozo B and Shetty S (2016) An investigation into suicides among Bhutanese refugees resettled in the United States between 2008 and 2011. Journal of Immigrant and Minority Health 18(4), 819–827. 10.1007/s10903-015-0326-6. 26758579 PMC4905799

[r21] Heinz A, Zhao X and Liu S (2020) Implications of the Association of Social Exclusion with Mental Health. JAMA Psychiatry 77(2), 113. 10.1001/jamapsychiatry.2019.3009. 31596429

[r22] Heredia Montesinos A (2015) Precipitating and risk factors for suicidal behavior among immigrants and ethnic minorities in Europe: A review of the literature. Suicidal Online. 4, 60–80.

[r23] Hovey JD and Magaña CG (2003) Suicide risk factors among Mexican migrant farmworker women in the Midwest United States. Archives of Suicide Research 7(2), 107–121. 10.1080/13811110301579.

[r24] ISSP Research Group (2015) International Social Survey Programme: National Identity III - ISSP 2013. Köln: GESIS Datenarchiv. ZA5950 Datenfile Version 2.0.0. 10.4232/1.12312.

[r25] Johnson CM, Rostila M, Svensson AC and Engström K (2017) The role of social capital in explaining mental health inequalities between immigrants and Swedish-born: A population-based cross-sectional study. BMC Public Health 17(1), 117. 10.1186/s12889-016-3955-3. 28122593 PMC5264487

[r26] Juang LP and Cookston JT (2009) Acculturation, discrimination, and depressive symptoms among Chinese American adolescents: A longitudinal study. The Journal of Primary Prevention 30(3–4), 475–496. 10.1007/s10935-009-0177-9. 19381814

[r27] Karasz A, Gany F, Escobar J, Flores C, Prasad L, Inman A, Kalasapudi V, Kosi R, Murthy M, Leng J and Diwan S (2019) Mental health and stress among south Asians. Journal of Immigrant and Minority Health 21(1), 7–14. 10.1007/s10903-016-0501-4. 27848078 PMC5643212

[r28] Karlsen S and Nazroo JY (2002) Relation between racial discrimination, social class, and health among ethnic minority groups. American Journal of Public Health 92(4), 624–631. 10.2105/ajph.92.4.624. 11919063 PMC1447128

[r29] Kessler RC, Berglund P, Demler O, Jin R, Koretz D, Merikangas KR, Rush AJ, Walters EE and Wang PS (2003) The epidemiology of major depressive disorder: Results from the National Comorbidity Survey Replication (NCS-R). JAMA 289(23), 3095–3105. 10.1001/jama.289.23.3095. 12813115

[r30] Kim A (2018) Social exclusion of multicultural families in Korea. Social Sciences 7(4), 63. 10.3390/socsci7040063.

[r31] Kim HM (2007) The state and migrant women: Diverging hopes in the making of “multicultural families” in contemporary Korea. Korea Journal 47(4), 100–122. 10.25024/kj.2007.47.4.100.

[r32] Kim MA, Ham OK, Cho I, Lee Ej and Lee BG (2022) Level of acculturation and acculturative stress perceived by Asian immigrant women married to south Korean men. Journal of Transcultural Nursing 33(1), 49–56. 10.1177/10436596211023977. 34130552

[r33] Kroenke K, Spitzer RL and Williams JB (2001) The PHQ-9: Validity of a brief depression severity measure. Journal of General Internal Medicine 16(9), 606–613. 10.1046/j.1525-1497.2001.016009606.x. 11556941 PMC1495268

[r34] Lazarus RS (1984) Stress, Appraisal, and Coping. New York: Springer.

[r35] Lusk M, Terrazas S, Caro J, Chaparro P and Puga Antúnez D (2021) Resilience, faith, and social supports among migrants and refugees from Central America and Mexico. Journal of Spirituality in Mental Health 23(1), 1–22. 10.1080/19349637.2019.1620668.

[r36] Migration Policy Institute (2024) U.S. Immigrant Population and Share over Time, 1850–Present. Available at http://migrationpolicy.org/programs/data-hub (accessed 24 June 2024).

[r37] Ministry of Gender Equality and Family (2022) A Survey on the Actual Conditions of Multicultural Families. Sejong: Ministry of Gender Equality and Family.

[r38] Ministry of Gender Equality and Family (2023a) 4th Master Plan for Multicultural Families Policy (2023–2027). Sejong: Ministry of Gender Equality and Family.

[r39] Ministry of Gender Equality and Family (2023b) Statistics Relating to Multicultural Family. Division MF (ed.). Sejong: Ministry of Gender Equality and Family.

[r40] Mohammadifirouzeh M, Oh KM, Basnyat I and Gimm G (2023) Factors associated with professional mental help-seeking among U.S. immigrants: A systematic review. Journal of Immigrant and Minority Health 25(5), 1118–1136. 10.1007/s10903-023-01475-4. 37000385 PMC10063938

[r41] Montemitro C, D’Andrea G, Cesa F, Martinotti G, Pettorruso M, Di Giannantonio M, Muratori R and Tarricone I (2021) Language proficiency and mental disorders among migrants: A systematic review. European Psychiatry 64(1), e49. 10.1192/j.eurpsy.2021.2224. 34315554 PMC8390337

[r42] Montesinos A, Heinz A, Schouler-Ocak M and Aichberger M (2013) Precipitating and risk factors for suicidal behaviour among immigrant and ethnic minority women in Europe: A systematic review. Suicidol Online 4, 60–80.

[r43] Nazroo JY (2003) The structuring of ethnic inequalities in health: Economic position, racial discrimination, and racism. American Journal of Public Health 93(2), 277–284. 10.2105/ajph.93.2.277. 12554585 PMC1447729

[r44] Ngwena J (2014) Black and minority ethnic groups (BME) suicide, admission with suicide or self-harm: An inner city study. Journal of Public Health 22(2), 155–163. 10.1007/s10389-013-0600-9.

[r45] Ornelas IJ and Perreira KM (2011) The role of migration in the development of depressive symptoms among Latino immigrant parents in the USA. Social Science & Medicine 73(8), 1169–1177. 10.1016/j.socscimed.2011.07.002. 21908089 PMC3185160

[r46] Park S, Lee M, Park SJ and Lee MG (2018) Health risk behaviors and psychological problems among south Korean, north Korean, and other multicultural family adolescents (2011–2016). Psychiatry Research 268, 373–380. 10.1016/j.psychres.2018.07.042. 30103182

[r47] Sanchez M, Diez S, Fava NM, Cyrus E, Ravelo G, Rojas P, Li T, Cano MA and De La Rosa M (2019) Immigration stress among recent Latino immigrants: The protective role of social support and religious social capital. Social Work in Public Health 34(4), 279–292. 10.1080/19371918.2019.1606749. 31033427 PMC9872174

[r49] Spallek J, Reeske A, Norredam M, Nielsen SS, Lehnhardt J and Razum O (2015) Suicide among immigrants in Europe—A systematic literature review. The European Journal of Public Health 25(1), 63–71. 10.1093/eurpub/cku121. 25096258

[r50] Statistics Korea (2023) Population Census. Daejeon: Statistics Korea.

[r51] Stein GL, Kiang L, Supple AJ and Gonzalez LM (2014) Ethnic identity as a protective factor in the lives of Asian American adolescents. Asian American Journal of Psychology 5(3), 206–213. 10.1037/a0034811.

[r52] Stronks K, Şekercan A, Snijder M, Lok A, Verhoeff AP, Kunst AE and Galenkamp H (2020) Higher prevalence of depressed mood in immigrants’ offspring reflects their social conditions in the host country: The HELIUS study. PLoS One 15(6), e0234006. 10.1371/journal.pone.0234006. 32497057 PMC7272005

[r53] Walsh SD, Edelstein A and Vota D (2012) Suicidal ideation and alcohol use among Ethiopian adolescents in Israel. European Psychologist 17(2), 131–142. 10.1027/1016-9040/a000115.

[r54] Wang L, Lin H-C and Wong YJ (2021) Perceived racial discrimination on the change of suicide risk among ethnic minorities in the United States. Ethnicity & Health 26(5), 631–645. 10.1080/13557858.2018.1557117. 30525981

[r55] Wong YJ, Vaughan EL, Liu T and Chang TK (2014) Asian Americans’ proportion of life in the United States and suicide ideation: The moderating effects of ethnic subgroups. Asian American Journal of Psychology 5(3), 237–242.

[r56] Wyatt LC, Ung T, Park R, Kwon SC and Trinh-Shevrin C (2015) Risk factors of suicide and depression among Asian American, native Hawaiian, and Pacific islander youth: A systematic literature review. Journal of Health Care for the Poor and Underserved 26(2), 191–237. 10.1353/hpu.2015.0059. 25981098 PMC4530970

[r57] Yeung W-JJ and Mu Z (2020) Migration and marriage in Asian contexts. Journal of Ethnic and Migration Studies 46(14), 2863–2879. 10.1080/1369183x.2019.1585005.

[r58] Yoo C (2021) Acculturation strategies of multi-cultural family adolescents in South Korea: Marginalization, separation, assimilation, and integration. International Journal of Intercultural Relations 81, 9–19. 10.1016/j.ijintrel.2020.12.011.

