# Supplementary material for: Mental health vulnerability in multicultural families: Risk factors among homogenous country
Source: Glob Ment Health (Camb). 2024 Oct 24;11:e101. doi: 10.1017/gmh.2024.108 (PMC11504928; doi:10.1017/gmh.2024.108)
Supplement: Peer review history

# Editor report: Recommendation: Mental health vulnerability in multicultural families: Risk factors among homogenous country — R0/PR2

**DOI:** 10.1017/gmh.2024.108.pr2

**Affiliations:** School of Public Health and Preventive Medicine, Monash University Faculty of Medicine Nursing and Health Sciences, Australia

No accompanying comment.

# Editor report: Decision: Mental health vulnerability in multicultural families: Risk factors among homogenous country — R0/PR3

**DOI:** 10.1017/gmh.2024.108.pr3

**Affiliations:** Johns Hopkins University Bloomberg School of Public Health, United States

No accompanying comment.

# Editor report: Recommendation: Mental health vulnerability in multicultural families: Risk factors among homogenous country — R1/PR5

**DOI:** 10.1017/gmh.2024.108.pr5

**Affiliations:** School of Public Health and Preventive Medicine, Monash University Faculty of Medicine Nursing and Health Sciences, Australia

No accompanying comment.

# Editor report: Decision: Mental health vulnerability in multicultural families: Risk factors among homogenous country — R1/PR6

**DOI:** 10.1017/gmh.2024.108.pr6

**Affiliations:** Johns Hopkins University Bloomberg School of Public Health, United States

No accompanying comment.

# Editor report: Recommendation: Mental health vulnerability in multicultural families: Risk factors among homogenous country — R2/PR8

**DOI:** 10.1017/gmh.2024.108.pr8

**Affiliations:** School of Public Health and Preventive Medicine, Monash University Faculty of Medicine Nursing and Health Sciences, Australia

No accompanying comment.

# Editor report: Decision: Mental health vulnerability in multicultural families: Risk factors among homogenous country — R2/PR9

**DOI:** 10.1017/gmh.2024.108.pr9

**Affiliations:** Johns Hopkins University Bloomberg School of Public Health, United States

No accompanying comment.
